# Use of ultrasound to estimate tracheal diameter in unclipped brachycephalic dogs: A pilot study

**DOI:** 10.1002/vro2.70018

**Published:** 2025-09-02

**Authors:** Emily Brady, Mike Herrtage, Isabella Harding, Jane Ladlow

**Affiliations:** ^1^ The Queen's Veterinary School Hospital University of Cambridge Cambridge UK; ^2^ Granta Veterinary Specialists Linton UK

## Abstract

**Background:**

A significant contributory factor to the brachycephalic obstructive airway syndrome index of bulldogs is the diameter of their tracheas. Bulldogs are predisposed to tracheal hypoplasia. A non‐invasive, financially reasonable and widely accessible screening test for tracheal diameter would be useful for assessing the most appropriate dogs to breed from within bulldog populations and may help in predicting results following upper airway surgery.

**Method:**

A prospective method comparison study involving 10 client‐owned brachycephalic dogs was conducted. Each patient underwent tracheal ultrasonography using a handheld ultrasound device (Butterfly IQ+) followed by extubated computed tomography (CT) scanning of the head and neck. Ultrasonographic tracheal measurements were compared with CT tracheal measurements and analysed for agreement, accuracy, and inter‐observer and intra‐observer repeatability.

**Results:**

Bland‒Altman analysis showed there was good agreement between the ultrasound and CT tracheal measurements; however, the 95% limits of agreement were wide (+0.43 and ‒0.29 cm), indicating that the ultrasound measurements lacked a high level of accuracy. Ultrasound in general overestimated the tracheal diameter by 0.07 cm (*p* < 0.05). Intra‐observer repeatability (mean range: 0.12 cm, average coefficient of variation [COV]: observer one;7.36%, observer two;5.53%, observer three;6.10%) was more consistent than inter‐observer repeatability (mean range: 0.26 cm, average COV: 8.47%).

**Conclusion:**

The accuracy of tracheal diameter measurements using an affordable handheld ultrasound device in unclipped brachycephalic patients was relatively low. However, technique modifications may significantly improve results, and further investigation is warranted to explore the utility of this method as a screening tool for tracheal hypoplasia in bulldog populations.

## INTRODUCTION

Brachycephalic breeds have been selectively bred over many years for their characteristic flat facial anatomy and foreshortened skulls. This selective breeding has led to significant alterations in upper airway conformation, resulting in turbulent and obstructed airflow, a condition known as brachycephalic obstructive airway syndrome (BOAS) in some brachycephalic patients.^1^ Key anatomical features contributing to BOAS include stenotic nares, an elongated and thickened soft palate, aberrant turbinates and a hypoplastic trachea, all of which create increased negative airway pressures during inspiration.^2^, ^3^ These heightened pressures can induce secondary changes, such as everted tonsils, everted laryngeal saccules, and laryngeal oedema and collapse, further exacerbating airway turbulence and obstruction.^2^, ^4^, ^5^

A BOAS index has been developed, which quantifies the severity of the upper airway obstruction in dogs with BOAS, where 0% equates to a non‐affected patient and 100% equates to the most severely affected individuals.^6^ This index allows an objective measurement to evaluate risk factors for BOAS and the effectiveness of surgical treatments. The most common breeds presenting for surgery include French bulldogs, pugs and bulldogs.^7^ Bulldogs are predisposed to hypoplastic tracheas, and while historical research has suggested that it did not affect prognosis after upper airway surgery and tracheal hypoplasia can be subclinical in the bulldog,^8^ recent research has found that narrow tracheal dimensions significantly effect their BOAS index.^9^ As this is an anatomical lesion that cannot surgically be improved, it is likely that it will also negatively impact their BOAS index following upper airway surgery. While the specific genetics of tracheal hypoplasia are not known, given the high occurrence of this disease within the bulldog population, a genetic component is assumed; hence, many veterinarians advise against breeding from patients with hypoplastic tracheas.

Tracheal assessment can be performed using multiple modalities, including computed tomography (CT),^10^, ^11^, ^12^ radiography,^12^, ^13^, ^14^ fluoroscopy^13^ and tracheoscopy.^15^, ^16^, ^17^ Each of these modalities carry their own advantages and disadvantages with respect to accessibility, financial cost, radiation exposure and the need for sedation or general anaesthesia. Radiography is likely the most widely accessible of these modalities and can be performed in most patients with only sedation. Tracheal diameter has been estimated within a population of beagle dogs with some success using radiography^14^; however, radiographic assessment of tracheal hypoplasia in English bulldog populations has been found to be inaccurate.^18^, ^19^ A non‐invasive and financially accessible screening test for tracheal diameter that can be performed on conscious patients would be valuable for identifying patients affected by tracheal hypoplasia, and advising against breeding from these individuals, and may also assist in predicting outcomes following upper airway surgery, given that tracheal hypoplasia cannot be surgically addressed. Ultrasonography is limited in its ability to fully visualise the whole trachea due to the presence of luminal air, which generates an acoustic shadow artefact that obscures the far side of the tracheal wall. Despite this limitation, ultrasonography has been utilised to assess tracheal collapse in canine patients,^20^, ^21^ and this artefact has also been employed to estimate the luminal diameter of the airway in both humans^22^ and a population of beagle dogs.^23^ Additionally, ultrasonography has been applied, with varying efficacy, to facilitate endotracheal tube selection in human patients.^24^, ^25^, ^26^, ^27^

The purpose of this study was to assess the agreement, accuracy, and inter‐observer and intra‐observer repeatability of ultrasonographic tracheal diameter measurements made using a handheld ultrasound device in unclipped, brachycephalic patients compared to measurements made using CT. If ultrasonography was found to estimate tracheal diameter accurately within this population, the author intends to conduct a follow‐up study to assess the screening capabilities of ultrasonography for tracheal hypoplasia in bulldogs.

## MATERIALS AND METHODS

As this is a diagnostic accuracy study, the standards for reporting diagnostic accuracy studies (STARD) guidelines^28^ were referred to and followed for reporting this study.

### Preliminary investigation

To verify the accuracy of the CT and ultrasound post‐processing measurements, a CT was performed on a cadaver prior to surgical removal of its trachea. The tracheal diameter was measured manually with a ruler and callipers to the nearest millimetre. The trachea was then submerged within a water bath and transverse images of the trachea were captured using the Butterfly IQ+ ultrasound probe. The CT and ultrasound images were both reviewed and were within 1 mm of agreement with the manual measurements. It was thus accepted that the CT and ultrasound post‐processing scales were consistent with the true scale.

### Subjects

The study was a prospective, method comparison study. Ethical approval was obtained from the Department of Veterinary Medicine at the University of Cambridge prior to commencement of the study. Patients were included if they belonged to a brachycephalic breed and received dental treatment at The Queen's Veterinary School Hospital, Cambridge, between June 2022 and September 2022. Patients were excluded if they had a history of airway surgery.

### Clinical procedures and image acquisition

Tracheal ultrasonography was performed in all patients immediately prior to induction of general anaesthesia for head and neck CT and subsequent dental treatment. In some cases, light premedication was administered before the ultrasound to facilitate anaesthetic induction; however, all patients remained conscious during the ultrasound examination. Premedication and general anaesthetic protocols were individualised based on each patient's clinical examination and medical history, resulting in variation between cases.

#### Ultrasonography

The patients were all assessed while in a sitting position with their neck slightly extended. All exams were performed using a portable ultrasound probe (Butterfly IQ+, linear transducer; 1‒10 MHz) attached to an iPad screen. The ultrasound probe was placed centrally on their ventral neck, perpendicular to the long axis of the trachea, approximately 2 cm caudal to the cricoid cartilage of the larynx. The patients were not clipped. Spirit and ultrasound gel (Aquason, Parker Laboratories BV) were used to improve contact between the probe and the skin. Between 5 and 10 images were acquired of the trachea of each patient.

#### Computed tomography

CT was performed immediately following ultrasonography. Patients were induced with propofol prior to intubation. Anaesthesia was maintained using oxygen and isoflurane. The patients were extubated, and CT of the head and neck was performed with the patients in sternal recumbency. CT was performed with a 16‐slice CT scanner (Aquilion 16, Toshiba America Medical Systems). The images were acquired in helical mode with slice thickness of 0.5 mm, a pitch factor of 0.938 and a 512 × 512 matrix. The images were acquired with a bone algorithm (window width [WW]: 3500 Hounsfield units [HU]; window level [WL]: 1000 HU) and soft tissue algorithm (WW: 350 HU; WL: 35 HU). Following the CT, the patients were reintubated and underwent the dental procedure.

### Tracheal diameter measurements

**FIGURE 1:**
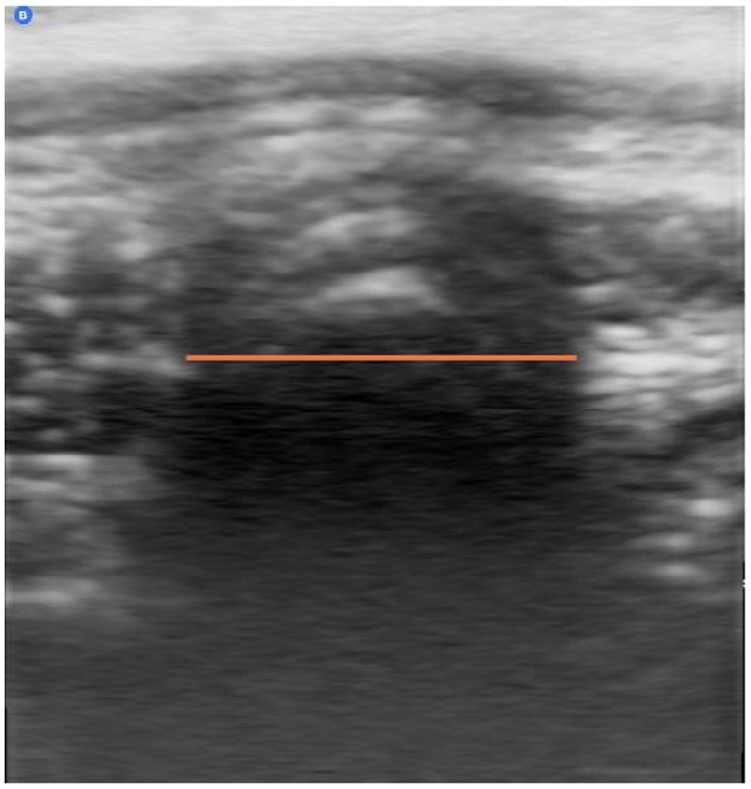
Ultrasonographic image of the trachea in transverse. The tracheal diameter was measured as the distance between the air‒mucosa interface margins on each side of the trachea; the acoustic shadow (orange line).

Three observers of differing levels of expertise (observer one: European College of Veterinary Surgeons diplomate; observer two: rotating intern; observer three: European College of Veterinary Diagnostic Imaging and European College of Veterinary Internal Medicine diplomate) were recruited. The transverse tracheal diameter was estimated by measuring the acoustic shadow in each image, which was created by the air‒mucosa interface (Figure 1). Each observer was given a short training session on how to interpret the ultrasound images and recognise the acoustic shadow prior to commencing the study. From the ultrasound images acquired, 32 images were selected from the 10 patients for review. Images were excluded where there was no clear acoustic shadow. Each selected image was randomly allocated a number from 1 to 32. The observers were blinded to all subject details. The observers reviewed each figure and measured the acoustic shadow. To test intra‐observer repeatability, the images were randomised again, and all observers re‐measured the acoustic shadow after a 4‐week hiatus.

Ultrasound image analysis was performed using Butterfly Cloud v222.0.0.

**FIGURE 2:**
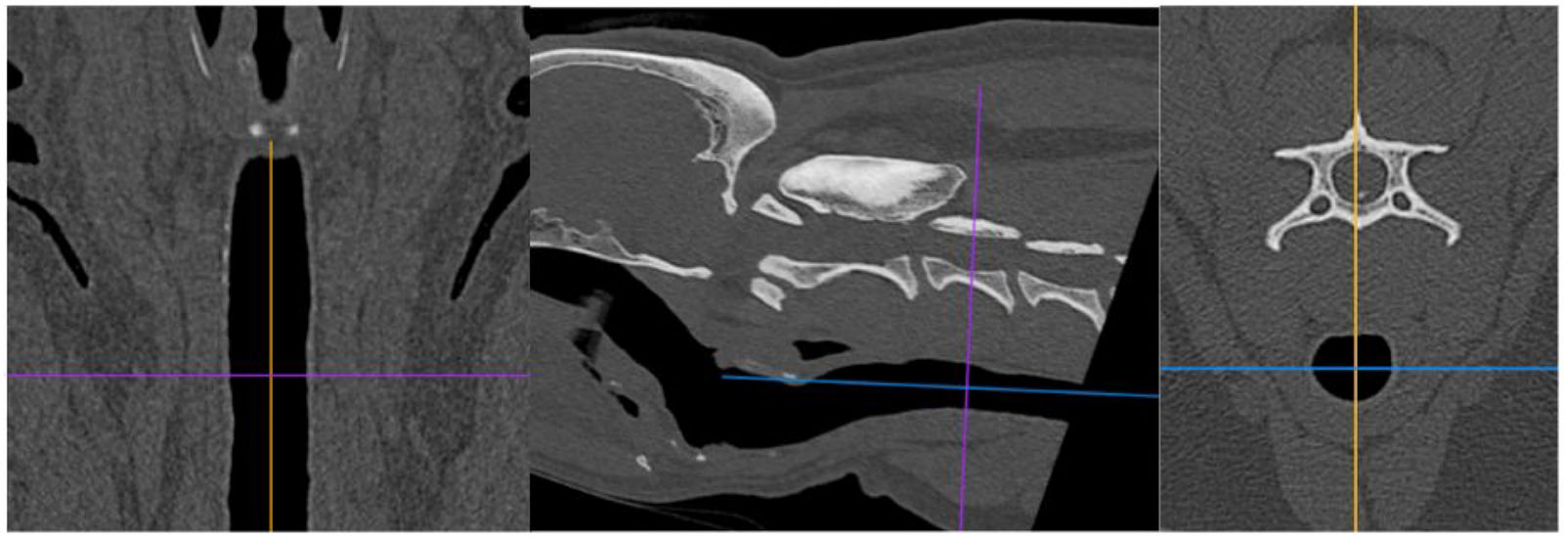
Computed tomography multiplanar reconstruction using a bone window (window width: 3500; window level: 1000) showing where the tracheal diameter was measured. The axis lines were positioned so that they dissect the trachea into four equal quarters. On the sagittal view, the ventrodorsal axis bisects the third cervical vertebrae. The trachea was measured within the transverse plane along the blue axis, from inside wall to inside wall.

CT image analysis was performed using commercially available software (Horos Project, version 3.3.6) using a bone window (WW: 3500 HU; WL: 1000 HU) by two observers (observers one and four; an imaging intern). Multiplanar reconstruction was used to measure the transverse cranial cervical tracheal diameter at the level of the middle third cervical vertebrae (Figure 2), which equates to approximately the same level from which the ultrasound images were acquired from. The average of the measurements made by the two observers was used. All CT tracheal diameter measurements were performed in non‐intubated patients as intubation has been found to increase the tracheal diameter.^29^

### Statistical analysis

All the data were entered into a statistical spreadsheet. Statistical analysis was performed using GraphPad Prism software version 10.2.3 (GraphPad Software). Bland‒Altman plots were produced to assess the agreement between the CT tracheal diameter and ultrasound tracheal diameter measurements. To assess consistency, the coefficient of variation (COV) was calculated using the results from all observers, and for each individual observer, and compared.

## RESULTS

Ten brachycephalic dogs (five Cavalier King Charles Spaniel, four Chihuahua and one Boxer) met the study criteria during the recruitment period. The patients had a mean weight of 8.7 kg (range: 3.1‒26.4 kg) and mean age of 7.1 years (range: 4.2‒8.3 years). Five dogs were male neutered and five were female neutered.

**FIGURE 3:**
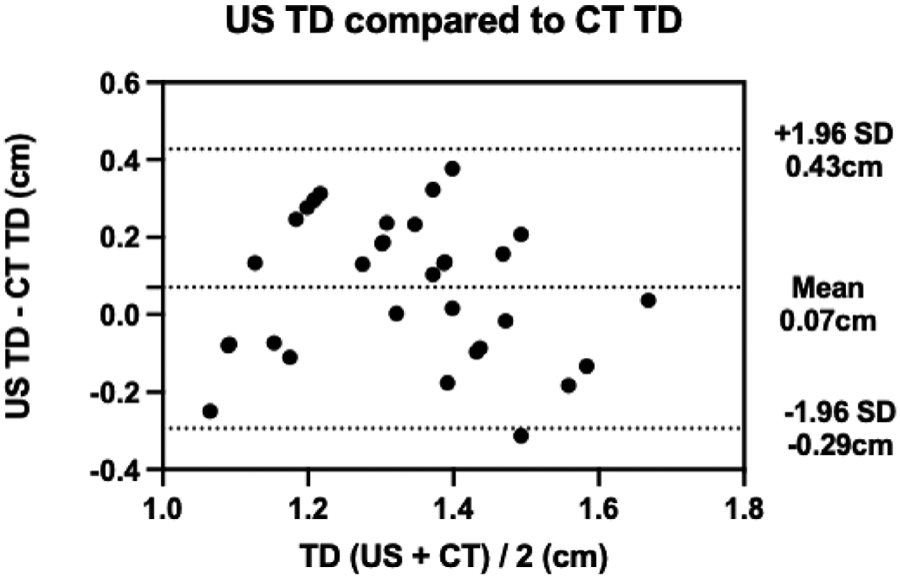
Bland‒Altman graph showing ultrasound (US) tracheal diameter (TD) measurements compared to computed tomography (CT) TD measurements. Dotted lines represent the means, and +1.96 and – 1.96 represent standard deviations (SDs).

As calculated by CT analysis, the diameter of the tracheas within this study ranged from 1.06 to 1.65 cm, with an average of 1.30 cm. Bland‒Altman analysis showed good agreement between CT tracheal diameter and ultrasound tracheal diameter measurements. However, using a paired *t*‐test a significant difference was found when comparing the ultrasound tracheal diameter measurements and the CT tracheal diameter measurements. There was a bias of +0.07 cm (*p*‐value of 0.049), indicating that ultrasonography in general overestimated the tracheal diameter. The 95% limits of agreement were +0.43 and ‒0.29 cm (Figure 3).

When comparing the average CT measurement with the average of each observer's ultrasound measurements, 90.6% (29/32) of differences were within +0.29 and ‒0.30 cm for observer one, within +0.23 and ‒0.30 cm for observer two and within +0.35 and ‒0.39 cm for observer three. Using the average of all three observer's ultrasound measurements, 87.5% (28/32) of the measurements were within +0.29 and ‒0.25 cm of the average of the CT measurement. The mean difference was +0.08 cm for observer one and +0.06 cm for observers two and three. The COV was calculated for each of the 32 images using the results of all three observers. From this, the average inter‐observer COV was calculated to assess the repeatability of the measurements made by all observers, which was considered good (8.47%, range: 1.94%‒22.67%).

With regards to intra‐observer repeatability, the intra‐observer range of measurements for the ultrasound images showed that 75% (24/32) of all measurements had a range of 0.27 cm or less. The mean range was 0.26 cm. The intra‐observer range showed that 84.4% (27/32) of observer one's measurements had a range of 0.28 cm or less, 96.9% (31/32) of observer two's measurements had a range of 0.2 cm or less, and 93.8% (30/32) of observer three's measurements had a range of 0.25 cm or less. The mean ranges were 0.13, 0.11 and 0.12 cm for observers one, two and three, respectively. The COV was calculated for each observer for each of the 32 images. From this, the average COV was calculated for each observer to assess repeatability. The average COV was considered good for all observers (observer one: 7.36%, range: 0.00%‒26.07%; observer two: 5.53%, range: 0.00%‒23.90%; observer three: 6.10%, range: 0.00%‒37.07%).

## DISCUSSION

The aim of this study was to assess the accuracy and repeatability of tracheal diameter measurements made using a portable ultrasound machine in conscious, unclipped brachycephalic patients. While Bland‒Altman analysis showed there was good agreement between the ultrasound and CT measurements, the 95% limits of agreement were wide: +0.43 and ‒0.29 cm. Given that the average tracheal diameter as measured using CT was 1.30 cm, the widely dispersed 95% limits of agreement equated to +33% and ‒22% of the total tracheal diameter; hence, ultrasonography was not highly accurate with this methodology in this population of patients. There was a significant bias of +0.07 cm (*p* < 0.05), indicating that in general ultrasound overestimated the tracheal diameter when compared to CT. This contrasts with Kim et al.,^23^ who found that in general ultrasound underestimated tracheal diameter. This may in part be due to intubation of the patients in the study by Kim et al.,^23^ as intubation has been found to increase tracheal width and height measurements by approximately 10% in a population of brachycephalic dogs.^29^ This is why we ensured that all patients were not intubated for the CT assessment within our study. We cannot rule out the possibility that prior intubation may have altered the tracheal dimensions (e.g., by tracheal irritation), although this is deemed unlikely to have statistically impacted the results. Another factor that may have contributed is tracheal flaccidity; chihuahuas, and to a lesser degree Cavalier King Charles Spaniels, are prone to tracheal collapse.^16^, ^30^ While none of the patients had a reported history consistent with clinical tracheal collapse, it was noted that four patients (all four chihuahua patients) on CT showed flaccidity of the dorsal tracheal ligament, resulting in luminal narrowing in a ventrodorsal direction. This flaccidity was most prominent within the thoracic inlet, and only minimally evident within the cranial cervical region. However, given the increased susceptibility within our population of patients to suffer from tracheal flaccidity, it is possible that pressure from the ultrasound probe may have partially flattened the trachea, widening the acoustic shadow and hence the tracheal diameter as recorded via ultrasonography. Given that our patients were not clipped for the ultrasound acquisition, this may have increased the probe pressure needed to acquire suitable images and potentially exacerbated tracheal flattening.

In our study, we found intra‐observer repeatability to be slightly more consistent than inter‐observer repeatability, as represented by each individual observer receiving a lower COV than when all observers's results were assessed together. While the average COV for both intra‐observer and inter‐observer repeatability was less than 10%, which is considered good, the authors consider the range to be relatively high, meaning that the interpretation of each individual image was not highly consistent.

While the public and breeders are becoming increasingly aware of BOAS and the welfare concern it poses, accessibility to screening regimes is important in order to increase uptake prior to consideration of animals for breeding. Therefore, this study was designed with the ultimate ambition of developing an accessible, minimally invasive and low‐cost screening method for assessing tracheal diameter of bulldogs prior to consideration for breeding or surgical airway intervention that could be replicated within a non‐referral setting when airway assessment via CT is not an option. The study was designed to complement the existing respiratory functional grading scheme, which is already in place and helps assess severity of disease and guide recommendations for surgery and breeding.^31^ Given that tracheal hypoplasia cannot be surgically addressed and is likely partially inherited, identifying patients who have tracheal hypoplasia prior to breeding or surgical intervention would be useful. Patients were not clipped for the procedure, as many owners are reluctant to consent to clipping, which could deter participation in the screening. To enhance probe‒skin contact, spirit and ultrasound gel were used; however, the decision to leave patients unclipped may have contributed to reduced image clarity. This, in turn, could have resulted in a less defined acoustic shadow and increased observer variability. A portable and relatively inexpensive ultrasound device was employed, reflecting the equipment typically available in most general veterinary practices, as opposed to a high‐end referral‐level ultrasound machine. The Butterfly probe connects to a wide range of display screens, including many smartphones and can be used in remote locations, which would facilitate the screening of dogs outside of veterinary clinics.

This study has some limitations. The patients studied were brachycephalic patients presenting for dental procedures and consequently were older in age and not the classic breed distribution presenting for BOAS surgery or tracheal hypoplasia. As stated previously, the patients included in this study were more prone to tracheal flaccidity, which may have impacted the accuracy of the ultrasonographic measurements. Further assessment is intended within the bulldog population, and we predict that the results may be improved given that bulldog tracheas are not prone to collapse.

Respiratory phase was not taken into account during either the CT or ultrasound. While one study showed changes in tracheal height during phases of respiration, the change in width within the cervical region was small (0.7%).^10^ Our patients often had a high respiratory rate during their ultrasound examination, and it was not possible to obtain images only during one phase of respiration. In addition, we felt that not controlling for these factors would be more representative of an assessment within a non‐referral setting.

To test repeatability, we used previously recorded images by a single sonographer, but we did not test inter‐operator variability associated with image acquisition and scan performance. The images were assessed at a time separate to acquisition, which can often make sonographic image interpretation more difficult. In a real‐life setting, it is likely that the person acquiring the images will be measuring the acoustic shadow at the time of acquisition, which may improve the accuracy of the results.

Only one tracheal site was measured, given that tracheal diameter can vary along its length,^32^, ^33^ it is possible that the cranial cervical tracheal diameter may not represent the narrowest tracheal site; however, this narrowing is often in a ventrodorsal direction, and bulldog populations are likely to be minimal given that they are not commonly affected by tracheal collapse. In the study by Kim et al.,^23^ who also compared ultrasound measurements with CT measurements within a population of laboratory beagle dogs, the thoracic inlet ultrasonographic measurements were found to differ more compared to the sublaryngeal ultrasonographic measurements.

In conclusion, we found that while ultrasonographic estimation of tracheal diameter shows good agreement with measurements obtained via CT, its accuracy in this study, which was designed to replicate a minimally invasive screening method using widely available resources on conscious, unclipped patients, was low. Inter‐observer and intra‐observer repeatability were considered good based on the average of the COV calculations; however, the range of results was high, meaning that the measurements for each individual image were not highly consistent. Therefore, ultrasonography may have restricted utility as a screening tool for tracheal hypoplasia via this method in affected populations. However, alterations to the technique used in this study may improve the results significantly and thus further investigations are warranted.

## AUTHOR CONTRIBUTIONS

Jane Francis Ladlow and Emily Brady designed the study. Jane Francis Ladlow, Mike E. Herrtage and Isabella Harding assessed the images to produce the data. Emily Brady acquired, analysed and interpreted the data. The manuscript was primarily written by Emily Brady, and reviewed and adjusted by Jane Francis Ladlow and Mike E. Herrtage.

## CONFLICTS OF INTEREST

The authors declare they have no conflicts of interest.

## ETHICS STATEMENT

Ethical approval was obtained from the Department of Veterinary Medicine Ethics Committee at the University of Cambridge prior to commencement of the study (ethics approval number: CR562). All dog owners provided informed consent prior to inclusion of their dogs in the study.

## Data Availability

The data that support the findings of this study are available from the corresponding author upon reasonable request.
